# Complicated intra-abdominal infections in Europe: a comprehensive review of the CIAO study

**DOI:** 10.1186/1749-7922-7-36

**Published:** 2012-11-29

**Authors:** Massimo Sartelli, Fausto Catena, Luca Ansaloni, Ari Leppaniemi, Korhan Taviloglu, Harry van Goor, Pierluigi Viale, Daniel Vasco Lazzareschi, Federico Coccolini, Davide Corbella, Carlo de Werra, Daniele Marrelli, Sergio Colizza, Rodolfo Scibè, Halil Alis, Nurkan Torer, Salvador Navarro, Boris Sakakushev, Damien Massalou, Goran Augustin, Marco Catani, Saila Kauhanen, Pieter Pletinckx, Jakub Kenig, Salomone Di Saverio, Elio Jovine, Gianluca Guercioni, Matej Skrovina, Rafael Diaz-Nieto, Alessandro Ferrero, Stefano Rausei, Samipetteri Laine, Piotr Major, Eliane Angst, Olivier Pittet, Ihor Herych, Ferdinando Agresta, Nereo Vettoretto, Elia Poiasina, Jaan Tepp, Gunter Weiss, Giorgio Vasquez, Nikola Vladov, Cristian Tranà, Samir Delibegovic, Adam Dziki, Giorgio Giraudo, Jorge Pereira, Helen Tzerbinis, David van Dellen, Martin Hutan, Andras Vereczkei, Avdyl Krasniqi, Charalampos Seretis, Cristian Mesina, Miran Rems, Fabio Cesare Campanile, Pietro Coletta, Mirjami Uotila-Nieminen, Mario Dente, Konstantinos Bouliaris, Konstantinos Lasithiotakis, Vladimir Khokha, Dragoljub Zivanovic, Dmitry Smirnov, Athanasios Marinis, Ionut Negoi, Ludwig Ney, Roberto Bini, Miguel Leon, Sergio Aloia, Cyrille Huchon, Radu Moldovanu, Renato Bessa de Melo, Dimitrios Giakoustidis, Orestis Ioannidis, Michele Cucchi, Tadeja Pintar, Zoran Krivokapic, Jelena Petrovic

**Affiliations:** 1 Department of Surgery, Macerata Hospital, Macerata, Italy; 2 Emergency Surgery, Maggiore Parma Hospital, Parma, Italy; 3 Department of General Surgery, Ospedali Riuniti, Bergamo, Italy; 4 Department of Abdominal Surgery, University Hospital Meilahti, Helsinki, Finland; 5 Department of Surgery, Sisli Florence Nigtingale Hospital, Istanbul, Turkey; 6 Department of Surgery, Radboud University Nijmegen Medical Centre, Nijmegen, Netherlands; 7 Department of Internal Medicine Geriatrics and Nephrologic Diseases, Clinic of Infectious Diseases, St Orsola-Malpighi University Hospital, Bologna, Italy; 8 Department of Anestesiology, Ospedali Riuniti, Bergamo, Italy; 9 General, Oncological, Geriatrical Surgery and advanced Technology, University Federico II, Naples, Italy; 10 Department of Human Pathology and Oncology, Policlinico le Scotte, University Hospital, Siena, Italy; 11 Department of Surgery, Fatebenefratelli Isola Tiberina hospital, Rome, Italy; 12 Department of General Surgery, Bakirkoy Training Research Hospital, Istanbul, Turkey; 13 Department of General Surgery, Baskent University Faculty of Medicine, Adana, Turkey; 14 Department of Surgery, Parc Tauli University Hospital, Barcelona, Spain; 15 First General Surgery Clinic, University Hospital St. George/Chair of Surgical Propedeutics, Medical Faculty, Medical University Plovdiv, Plovdic, Bulgaria; 16 Department of Emergency Surgery, University Hospital of Nice, University of Nice Sophia-Antipolis, Sophia-Antipolis, France; 17 Department of Surgery, University Hospital Center, Zagreb, Croatia; 18 Emergency Department, Umberto I Hospital, “La Sapienza” University of Rome, Rome, Italy; 19 Department of Gastroenterological surgery Turku, University Central Hospital, Turku, Finland; 20 Department of Surgery, AZ Maria Middelares, Ghent, Belgium; 21 3rd Department of Generał Surgery, Narutowicz Hospital, Krakow, Połand; 22 Department of Surgery, Maggiore Hospital, Bologna, Italy; 23 Department of Surgery, Mazzoni Hospital, Ascoli Piceno, Italy; 24 Department of Surgery Hospital and Oncological Centre Novy Jicin, Novy Jicin, Czech republic; 25 Department of General and Digestive Surgery, Virgen de la Victoria University Hospital, Malaga, Spain; 26 Department of Surgery, Mauriziano Hospital, Torino, Italy; 27 Department of Surgery (Chief Renzo Dionigi), University of Insubria, Ospedale di Circolo e Fondazione Macchi, Varese, Italy; 28 Department GI-surgery, Kuopio University Hospital, Kuopio, Finland; 29 2nd Department of Surgery, Jagiellonian University, Krakow, Poland; 30 Department of Visceral Surgery and Medicine, Inselspital Bern, University of Bern, Bern, Switzerland; 31 Department of Visceral Surgery Centre Hospitalier Universitaire Vaudois, CHUV, Lausanne, Switzerland; 32 Department of General Surgery, Lviv Emergency Hospital, Lviv, Ukraine; 33 Department of General Surgery, Ospedale Civile, Adria (RO), Italy; 34 General and Vascular Surgery, M.Mellini Hospital, Chiari, Italy; 35 First General Surgery, North Estonia Regional Hospital, Tallinn, Estonia; 36 Intensive Care Klinikum, Magdeburg gGmbH, Magdeburg, Germany; 37 Department of Emergency Surgery Azienda Ospedaliero-Universitaria S.Anna, Ferrara, Italy; 38 Department of Hepato-biliary and Pancreatic surgery and Transplantology, Military Medical Hospital, Sofia, Bulgaria; 39 Department of Surgery, Ospedali Riuniti Umberto I-Lancisi-Salesi, Ancona, Italy; 40 Department of surgery, University Clinic Center Tuzla, Tuzla, Bosnia and Herzegovina; 41 Department of General and Colorectal Surgery, University Hospital, Central Veterans Hospital, Lodz, Poland; 42 Surgical Department Santa Croce e Carle hospital, Cuneo, Italy; 43 Department of Surgery, São Teotónio Hospital, Viseu, Portugal; 44 Department of HPB and Liver Transplant Surgery, Royal Free Hospital, London, United Kingdom; 45 Department of Surgery, Manchester Royal Infirmare, Manchester, UK; 46 IInd Surgical department of Medical faculty Comenius University, University Hospital Bratislava, st. Cyril and Methodius Hospital, Bratislava, Slovakia; 47 Department of Surgery, Medical School University of Pécs, Pécs, Hungary; 48 Department of Abdominal Surgery, University Clinical Centre of Kosovo, Prishtina, Kosovo; 49 2nd Department of Surgery, General Army Hospital of Athens, Athens, Greece; 50 Second Surgical Clinic, Emergency Hospital of Craiova, Craiova, Roumanie; 51 Surgical Department, General hospital Jesenice, Jesenice, Slovenia; 52 Department of surgery, Andosilla Hospital, Civita Castellana, Italy; 53 Department of Surgery, Jesi Hospital, Jesi, Italy; 54 Department of Gastrointestinal Surgery, North Carelian Central Hospital, Joensuu, Finland; 55 Oncologic, Digestive and Emergency Surgery, Bocage Hospital, Dijon, France; 56 Surgical Department General Hospital of Larissa, Larissa, Greece; 57 Department of General Surgery, University Hospital of Heraklion, Heraklion, Greece; 58 Surgical Department, Mozyr, Belarus; 59 Department of Pediatric surgery, Paediatric surgery and orthopaedic Clinic, Nis, Serbia; 60 General Surgery, Clinical Hospital at Chelyabinsk Station OJSC "Russian Railroads", Chelyabinsk City, Russian Federation; 61 First Department of Surgery, Tzanion General Hospital, Piraeus, Greece; 62 Department of General Surgery, Emergency Hospital of Bucharest, Bucharest, Romania; 63 Deparment of Surgery, Downtown Campus, University Hospital of Munich, Munich, Germany; 64 General and emergency surgery, SG Bosco Hospital, Torino, Italy; 65 Department of General Surgery, Hospital La Paz, Madrid, Spain; 66 Department of Gynecology and Obstetrics, CHI Poissy-St-Germain-En-Laye, France and University Versailles Saint-Quentin, Versailles, France; 67 Chirurgie Viscerale, Digestive et Oncologique Hospital Prive, Arras les Bonnettes, Arras, France; 68 Department of General Surgery, Hospital São João Porto, Porto, Portugal; 69 Division of Transplantation, Department of Surgery, Medical School, Aristotle University of Thessaloniki, Hippokration General Hospital, Thessaloniki, Greece; 70 1st Surgical Department, General Regional Hospital "George Papanikolaou", Thessaloniki, Greece; 71 Department of Abdominal Surgery, umc Ljubljana, Ljubljana, Slovenia; 72 First Surgical clinic, Clinical Center of Serbia, School of Medicine, University of Belgrade 8, Belgrade, Serbia

## Abstract

The CIAO Study (“*C*omplicated *Intra-A*bdominal infection *O*bservational” Study) is a multicenter investigation performed in 68 medical institutions throughout Europe over the course of a 6-month observational period (January-June 2012).

Patients with either community-acquired or healthcare-associated complicated intra-abdominal infections (IAIs) were included in the study.

2,152 patients with a mean age of 53.8 years (range: 4–98 years) were enrolled in the study. 46.3% of the patients were women and 53.7% were men. Intraperitoneal specimens were collected from 62.2% of the enrolled patients, and from these samples, a variety of microorganisms were collectively identified.

The overall mortality rate was 7.5% (163/2.152).

According to multivariate analysis of the compiled data, several criteria were found to be independent variables predictive of patient mortality, including patient age, the presence of an intestinal non-appendicular source of infection (colonic non-diverticular perforation, complicated diverticulitis, small bowel perforation), a delayed initial intervention (a delay exceeding 24 hours), sepsis and septic shock in the immediate post-operative period, and ICU admission.

Given the sweeping geographical distribution of the participating medical centers, the CIAO Study gives an accurate description of the epidemiological, clinical, microbiological, and treatment profiles of complicated intra-abdominal infections (IAIs) throughout Europe.

## Introduction

Intra-abdominal infections (IAIs) include a wide spectrum of pathological conditions, ranging from uncomplicated appendicitis to fecal peritonitis.

In the event of complicated IAI [1], the infection proceeds beyond a singularly affected organ and causes either localized peritonitis (intra-abdominal abscesses) or diffuse peritonitis. Effectively treating patients with complicated intra-abdominal infections involves both source control and antimicrobial therapy [2,3].

### Study design

**Figure 1:**
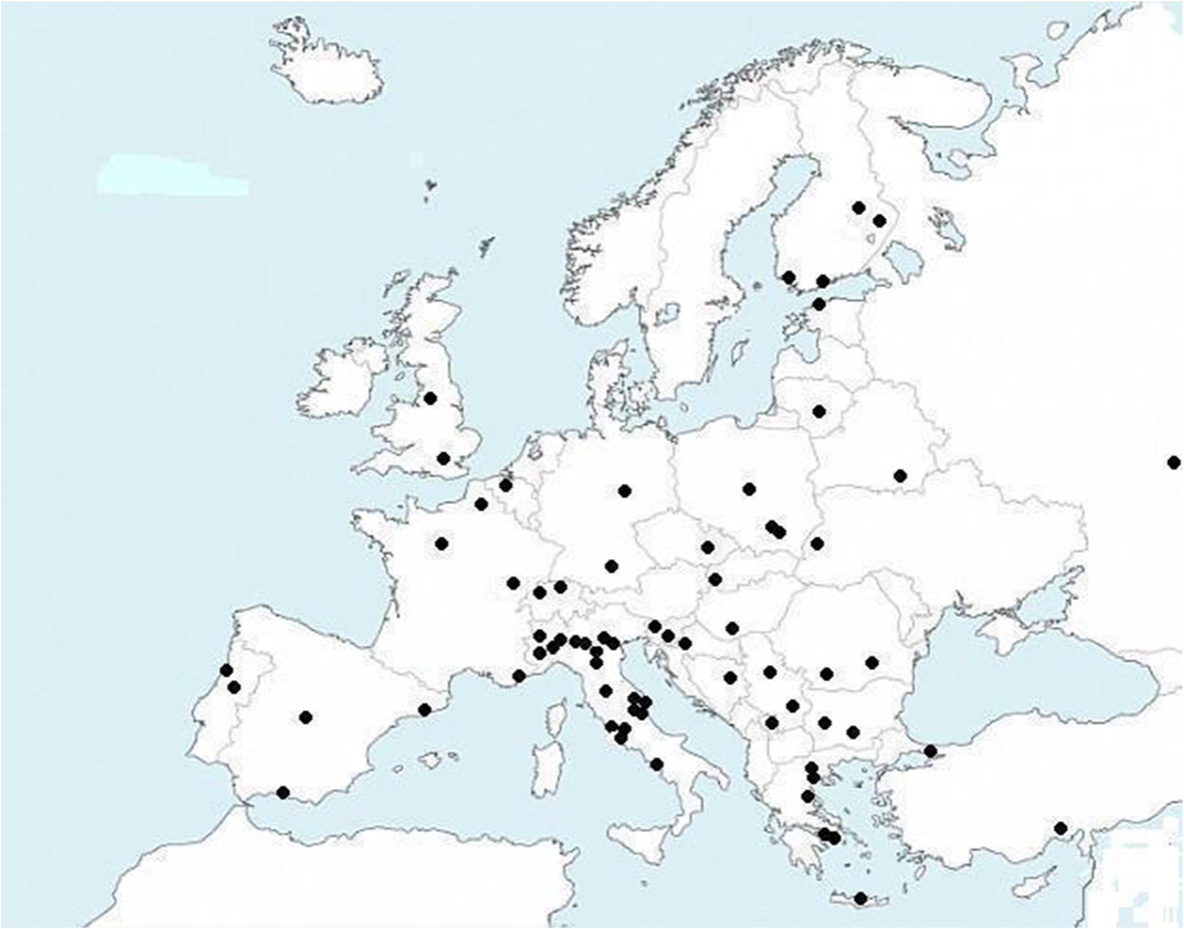
Geographic distribution of the CIAO Study.

The aim of the CIAO Study was to describe the epidemiological, clinical, microbiological, and surgical treatment profiles of community-acquired and healthcare-associated complicated intra-abdominal infections (IAIs) based on data collected over a 6-month period (January-June 2012) from 68 medical institutions throughout Europe (see Figure 1).

Patients with either community-acquired or healthcare-associated complicated intra-abdominal infections (IAIs) were included in the study.

The center coordinator of each participating medical institution collected and compiled clinical data in an online case report database.

The collected data included the following: (i) patient and disease characteristics, i.e. patient demographic data, type of infection (nosocomial or community-acquired), severity criteria, and previous antibiotic therapy administered in the 7 days preceding surgery; (ii) origin of infection, surgical procedures performed, and antibiotic therapies administered; and (iii) microbiological data, i.e. identification of bacteria and microorganismal pathogens within the peritoneal fluid, the identification of yeasts (if present), and the antibiotic susceptibilities of bacterial isolates.

This observational study did not attempt to change or modify the laboratory or clinical practices of the participating physicians or their respective institutions, and it did not require informed consent or formal approval by an Ethics Committee.

A Scientific Committee was established to impartially assess the objectives, methodology, and overall scientific quality of the project.

The study was monitored by the coordination center, which processed and verified missing or unclear data submitted to the central database.

Statistical analysis was performed using STATA® statistical software.

## Results

### Patients

2,152 patients with a mean age of 53.8 years (range 4–98) were enrolled in the CIAO Study. 996 patients (46.3%) were women and 1,156 (53.7%) were men. Among these patients, 1,701 (79%) were affected by community-acquired IAIs while the remaining 451 (21%) suffered from heathcare-associated infections. Intraperitoneal specimens were collected from 1,338 (62.2%) of the enrolled patients.

787 patients (36.5%) were affected by generalized peritonitis while 1,365 (63.5%) suffered from localized peritonitis or abscesses.

282 patients (13.1%) were admitted in critical condition (severe sepsis/septic shock).

Tables 1, 2 overviews the clinical findings and radiological assessments recorded upon patient admission.

**Table 1:** Clinical Findings

**Clinical findings**	**Patients**
	**n° (%)**
Abdominal pain	271 (12.6)
Abdominal pain, abdominal rigidity	192 (8.9%)
Abdominal pain, abdominal rigidità, T>38°C or <36°C, WBC >12,000 or < 4,000	366 (17%)
Abdominal pain, abdominal rigidity, T>38°C or <36°C,	70 (3.2)
Abdominal pain, abdominal rigidity, WBC >12,000 or < 4,000	445 (20.7%)
Abdominal pain, T>38°C or <36°C,	71 (3.3%)
Abdominal pain, T>38°C or <36°C, WBC >12,000 or < 4,000	235 (10.9%)
Abdominal pain, WBC >12,000 or < 4,000	325 (15.1)
T>38°C or <36°C	15 (0.7 %)
T>38°C or <36°C, WBC >12,000 or < 4,000	45 (2.0%)
Abdominal rigidity, WBC >12,000 or < 4,000	15 (0.7%)
Abdominal rigidity	15 (0.7%)
Abdominal rigidity, T>38°C or <36°C	22 (1%)
WBC >12,000 or < 4,000	32 (1.5%)
Not reported	33 (1.5%)

**Table 2:** Radiological Procedures

**Radiological procedures**	**Patients**
	**n° (%)**
Abdomen X ray	198 (9.2%)
Abdomen X ray, CT	164 (7.6%)
Abdomen X ray, ultrasound	401(18.6%)
Abdomen X ray, ultrasound, CT	205 (9.5%)
Abdomen X ray, ultrasound, MRI	3 (0.1%)
CT	527 (24.5%)
Ultrasound	345 (16.0%)
Ultrasound, CT	160 (8.3%)
Ultrasound, CT, MRI	5 (0.2%)
Ultrasound, MRI	6 (0.3%)
Not reported	131 (6%)

### Source control

The various sources of infection are outlined in Table 3. The most frequent source of infection was acute appendicitis; 798 cases (37%) involved appendicitis.

**Table 3:** Source of Infection

**Source of infection**	**Patients**
	**N 2152° (100%)**
Appendicitis	798 (37%)
Cholecystitis	289 (13.4%)
Post-operative	342 (15.,9%)
Colonic non diverticular perforation	158 (7.3%)
Gastroduodenal perforations	156 (7.3%)
Diverticulitis	166 (7.7%)
Small bowel perforation	103 (4.8%)
Others	110 (5.1%)
PID	18 (0.8%)
Post traumatic perforation	12 (0.6%)

The open appendectomy was the most common means of addressing complicated appendicitis. 443 patients (55.1%) admitted for complicated appendicitis underwent open appendectomies: 343 patients (77.4%) for localized infection or abscesses and 100 patients (29.1%) for generalized peritonitis. A laparoscopic appendectomy was performed for 318 patients (39.8%) with complicated acute appendicitis; of these patients, 217 underwent the procedure for localized peritonitis/abscesses and 101 underwent the procedure for generalized peritonitis. Open bowel resection was performed for 7 patients affected by complicated appendicitis. In the other 30 cases of complicated appendicitis (4.3%), conservative treatment (percutaneous drainage, surgical drainage, and non-operative treatment) was performed. 1.6% of patients underwent percutaneous drainage and interval appendectomies to address appendicular abscesses.

Among the patients with complicated cholecystitis (289), the open cholecystectomy was the most frequently performed procedure. 48.4% and 40.8% of cholecystitis patients underwent open and laparoscopic cholecystectomies, respectively. The remaining patients were treated with conservative methods (percutaneous drainage, non-operative treatment).

Among the patients with complicated diverticulitis (166) the Hartmann resection was the most frequently performed procedure. 73 patients (43.2%) underwent a Hartmann resection, and of these resections, the vast majority were open procedures (94.5% open compared to 5.5% laparoscopic). 54 of these patients (74%) underwent a Hartmann resection for generalized peritonitis, while the remaining 19 (26%) underwent the same procedure for localized peritonitis or abscesses. Colo-rectal resection was performed in 41 cases (24.7%). Laparoscopic resection was performed for only 3 patients (2 patients with and 1 patient without protective stoma) while open resection was performed for 38 patients (27 with and 11 without protective stoma).

The remaining patients received conservative treatment (percutaneous drainage, non-operative treatment, surgical drainage and stoma). 11 patients underwent laparoscopic drainage.

For patients with gastro-duodenal perforations (156 cases), the most common surgical procedure was gastro-duodenal suture. 107 patients underwent open gastro-duodenal suture (68.6%) and 18 patients underwent laparoscopic gastro-duodenal suture (11.5%). 16 patients (10.3%) underwent gastro-duodenal resection and 16 patients (10.3%) received conservative treatment (non-operative treatment, surgical drainage). The remaining patients underwent alternative procedures.

Of the 100 patients with small bowel perforations, 83 underwent open small bowel resection (83%) and 3 (3%) underwent laparoscopic small bowel resection. The remaining 14 patients (14%) were treated non-surgically.

Among the 158 patients with colonic non-diverticular perforation, 52 (32.9%) underwent open Hartmann resection, 55 (34.8%) underwent open resection with anastomosis and without stoma protection, and 23 underwent open resection with stoma protection (14.6%).

369 cases (17.1%) were attributable to post-operative infections. Anastomotic leaks were the most prevalent cause of post-operative infection. Of all post-operative infections, 40.2% resulted from colo-rectal leaks, 32.1% from upper gastro-intestinal leaks, 14.5% from biliary leaks, 11.2% from pancreatic leaks, and 1.9% from urinary leaks.

Source control was successfully implemented for 1,985 patients (92%) and proved ineffective for 167 patients (8%).

### Microbiology

Intraperitoneal specimens were collected from 1,339 patients (62.2%).

These specimens were obtained from 977 of the 1,701 patients presenting with community-acquired intra-abdominal infections (57.4%).

Intraperitoneal specimens were collected from 362 (80.3%) of the remaining 451 patients with nosocomial intra-abdominal infections.

The major pathogens involved in intra-abdominal infections were found to be *Enterobacteriaceae.*

The aerobic bacteria identified in samples of peritoneal fluid are reported in Table 4.

**Table 4:** Aerobic bacteria identified in peritoneal fluid

**Total**	**1,525 (100%)**
**Aerobic Gram-negative bacteria**	**1,041 (69.2%)**
Escherichia coli	632 (41.4%)
(Escherichia coli resistant to third generation cephalosporins)	64 (4.2%)
Klebsiella pneuumoniae	109 (7.1%)
(Klebsiella pneumoniae resistant to third generation cephalosporins)	37 (2.4%)
Enterobacter	63 (4.1%)
Proteus	33 (2.1 %)
Pseudomonas	80 (5.2%)
Others	124 (8.1%)
**Aerobic Gram-positive bacteria**	**484 (31.7%)**
Enterococcus faecalis	169 (11%)
Enterococcus faecium	72 (4.7%)
Staphylococcus Aureus	56 (3.7%)
Streptococcus spp.	100 (6,6%)
Others	87 (5.7%)

In community-acquired IAIs, Extended-Spectrum Beta-Lactamase (ESBL)-producing *Escherichia coli* isolates comprised 10.1% (64/632) of all *Escherichia coli* isolates, while ESBL-positive *Klebsiella pneumoniae* isolates represented 33.9% (37/109) of all *Klebsiella pneumoniae* isolates.

ESBL-positive *Enterobacteriaceae* were more prevalent in patients with nosocomial IAIs than they were in patients with community-acquired IAIs. ESBL-positive *Escherichia coli* isolates comprised 22.4% (34/152) of all identified *Escherichia coli* isolates, while ESBL-positive *Klebsiella pneumoniae* isolates made up 50% (26/52) of all identified *Klebsiella pneumoniae* isolates.

There were 5 isolates of *Klebsiella pneumoniae* resistant to Carbapenems. All Carbapenem-resistant *Klebsiella pneumoniae* isolates were acquired in an intensive care setting.

Among the identified aerobic gram-negative isolates, there were 80 isolates of *Pseudomonas aeruginosa*, comprising 5.3% of all identified aerobic bacteria isolates (4.3% in patients with community-acquired infections versus 6.7% in patients with nosocomial infections).

The 3 *Pseudomonas aeruginosa* strains resistant to Carbapenems were also obtained from nosocomial infections.

Among the identified aerobic gram-positive bacteria, *Enterococci* (*E. faecalis and E. faecium*) were the most prevalent, representing 16% of all aerobic isolates, and were identified in 241 cases. 22 glycopeptide-resistant *Enterococci* were identified; 16 were glycopeptide-resistant *Enterococcus faecalis* isolates and 6 were glycopeptide-resistant *Enterococcus faecium* isolates.

Although *Enterococci* were also present in community-acquired infections, they were far more prevalent in nosocomial infections.

Identified bacterial isolates from peritoneal fluid samples in both nosocomial and community-acquired IAIs are listed in Table 5.

**Table 5:** Aerobic bacteria in community-acquired and healthcare-associated (nosocomial) IAIs

**Community-acquired IAIs**	**Isolates**	**Healthcare-associated (nosocomial) IAIs**	**Isolates**
	**n°**		**n°**
Aerobic bacteria	988 (100%)	Aerobic bacteria	567 (100%)
Escherichia coli	480 (48.6%)	Escherichia coli	152 (26.8%)
(Escherichia coli resistant to third generation cephalosporins)	30 (3%)	(Escherichia coli resistant to third generation cephalosporins)	34 (6%)
Klebsiella pneumoniae	52 (5.2%)	Klebsiella pneumoniae	57 (10%)
(Klebsiella pneumoniae resistant to third generation cephalosporins)	11 (1,7%)	(Klebsiella pneumoniae resistant to third generation cephalosporins)	22 (6.7%)
Pseudomonas	42 (4.2%)	Pseudomonas	38 (6.7%)
Enterococcus faecalis	78 (7.9%)	Enterococcus faecalis	91 (16%)
Enterococcus faecium	39 (3.9%)	Enterococcus faecium	43 (7.6%)

Tests for anaerobes were conducted for 680 patients.

197 anaerobes were observed. The most frequently identified anaerobic pathogen was *Bacteroides*. 126 *Bacteroides* isolates were observed during the course of the study. Among the *Bacteroides* isolates, there were 3 Metronidazole-resistant strains.

Identified anaerobic bacteria are reported in Table 6.

**Table 6:** Anaerobic bacteria identified in peritoneal fluid

**Anaerobes**	**197**
Bacteroides	126 (64%)
(Bacteroides resistant to Metronidazole)	4 (2%)
Clostridium	16 (8.1%)
(Clostridium resistant to Metronidazole)	1 (0.5%)
Others	55 (27.9%)

Additionally, 138 *Candida* isolates were collectively identified (4.7%). 110 were *Candida albicans* and 28 were *non-albicans Candida*. 2 *Candida albicans* isolates and 7 *non-albicans Candida* isolates were resistant to Fluconazole (see Table 7).

**Table 7:** Candida isolates identified in peritoneal fluid

**Candida**	**138**
Candida albicans	110 (79.7%)
(Candida albicans resistant to Fluconazole)	4 (2.9%)
Non-albicans Candida	28 (20.3%)
(non-albicans Candida resistant to Fluconazole)	5 (3.6%)

### Outcome

The overall mortality rate was 7.6% (163/2,152).

521 patients (24.2%) were admitted to the intensive care unit in the early recovery phase immediately following surgery.

255 post-operative patients (11.8%) ultimately required additional surgeries; 66.7% of follow-up laparotomies were unplanned “on-demand” procedures and 20% were anticipated surgeries. Overall, 11.3% of these patients underwent open abdominal procedures.

According to univariate statistical analysis of the data (Table 8), severe sepsis (OR=14.6; 95%CI=8.7-24.4; p<0.0001) and septic shock (OR=27.6; 95%CI=15.9-47.8; p<0.0001) upon hospital admission were both predictive of patient mortality.

**Table 8:** Univariate analysis: risk factors for occurrence of death during hospitalization

**Risk factors**	**Odds ratio**	**95%CI**	**p**
*Clinical condition upon hospital admission*			
Severe sepsis	27.6	15.9-47.8	<0.0001
Septic shock	14.6	8.7-24.4	<0.0001
*Healthcare associated infection*			
Chronic care setting acquired	5.2	1.7-8.4	<0.0001
Non post-operative hospital acquired	3.8	2.4-10.9	<0.0001
Post-operative	2.5	1.7-3.7	<0.0001
*Source of infection*			
Colonic non diverticular perforation	117.4	27.9-493.9	<0.0001
Diverticulitis	45.4	10.4-198.6	<0.0001
Small bowel perforation	125.7	29.1-542	<0.0001
Delayed initial intervention	2.6	1.8-3.5	<0.0001
*Immediate post-operative clinical course*			
Severe sepsis	33.8	19.5-58.4	<0.0001
Septic shock	59.2	34.4-102.1	<0.0001
ICU admission	18.6	12-28.7	<0.0001
WBC>12000 or <4000 (3nd post-operative day)	2.8	1.8-4.4	<0.0001
T>38°C or <36°C (3nd post-operative day)	3.3	2.2-5	<0.0001

For healthcare associated infections, the setting of acquisition was also a variable found to be predictive of patient mortality (chronic care setting: OR=5.2; 95%CI=1.7-8.4; p<0.0001, non-operative hospital setting: OR=3.8; 95%CI=2.4-10.9; p<0.0001, and post-operative hospital setting: OR=2.5; 95%CI=1.7-3.7; p<0.0001).

Among the various sources of infection, colonic non-diverticular perforation (OR=117.4; 95%CI=27.9-493.9, p<0.0001), complicated diverticulitis (OR=45.4; 95%CI=10.4-198.6; p<0.0001), and small bowel perforation (OR=125.7; 95%CI=29.1-542; p<0.0001) were significantly correlated with patient mortality.

Mortality rates did not vary to a statistically significant degree between patients who received adequate source control and those who did not. However, a delayed initial intervention (a delay exceeding 24 hours) was associated with an increased mortality rate (OR=2.6; 95%CI=1.8-3.5; p<0.0001).

The nature of the immediate post-operative clinical period was a significant predictor of mortality (severe sepsis: OR=33.8; 95%CI=19.5-58.4; p<0.0001, septic shock: OR=59.2; 95%CI=34.4-102.1; p<0.0001). Patients requiring ICU admission (OR=18.6; 95%CI=12-28.7; p<0.0001) were also associated with increased mortality rates.

WBC counts greater than 12,000 or less than 4,000 (OR=2.8; 95%CI=1.8-4.4; p<0.0001), and core body temperatures greater than 38°C or less than 36°C (OR=3.3; 95%CI=2.2-5; p<0.0001) by the third post-operative day were significant predictors of patient mortality.

According to stepwise multivariate analysis (PR=0.005 and PE=0.001) (Table 9), several criteria were found to be independent variables predictive of mortality, including patient age (OR=3.3; 95%CI=2.2-5; p<0.0001), the presence of an intestinal non-appendicular source of infection (colonic non-diverticular perforation: OR=4.7; 95%CI=2.5-8; p<0.0001, complicated diverticulitis: OR=2.3; 95%CI=1.5-3.7; p<0.0001, small bowel perforation: OR=21.4; 95%CI=8-57.4; p<0.0001), a delayed initial intervention (a delay exceeding 24 hours) (OR=2.4; 95%CI=1.5-3.7; p<0.0001), severe sepsis (OR=6.6; 95%CI=3.8-11; P<0.0001) and septic shock (OR=7.2; 95%CI=4.12.5; p<0.0001) in the immediate post-operative period, and ICU admission (OR=3.8; 95%CI=2.2-6.4; p<0.0001).

**Table 9:** Multivariate analysis: risk factors for occurrence of death during hospitalization

**Risk factors**	**Odds ratio**	**95%CI**	**p**
Age	3.3	2.2-5	<0.0001
Severe sepsis in the immediate post-operative course	27.6	15.9-47.8	<0.0001
Septic shock in the immediate post-operative course	14.6	8.7-24.4	<0.0001
Colonic non diverticular perforation	4.7	2.5-8	<0.0001
Diverticulitis	2.3	1.5-3.7	<0.0001
Small bowel perforation	21.4	8-57.4	<0.0001
Delayed initial intervention	2.4	1.5-3.7	0.0001

Stepwise multivariate analysis, PR=0.005 E PE=0.001 (Hosmer-Lemeshow chi2(8)=1.68, area under ROC curve=0.9465).

## Discussion

### Source control

Complicated intra-abdominal infections are an important source of patient morbidity and are frequently associated with poor clinical prognoses, particularly for patients in high-risk categories.

The CIAO Study has confirmed that acute appendicitis is the most common intra-abdominal condition requiring emergency surgery in Europe. Both open and laparoscopic appendectomies are viable treatment options for complicated appendicitis [4]. The laparoscopic appendectomy is a safe and effective means of surgical treatment for addressing complicated intra-abdominal infections, but open surgery still retains several clinical advantages, including a reduced probability of post-operative intra-abdominal abscesses [5]. CIAO Study data indicate that the open approach was used in 55.1% of complicated appendicitis cases while the laparoscopic approach was performed in 39.8% of these cases.

For patients with periappendiceal abscesses, the proper course of surgical treatment remains a point of contention in the medical community. However, this contention notwithstanding, the most commonly employed treatment appears to be drainage with subsequent appendectomy [6].

Although guidelines for the management of intra-abdominal infections commonly assert that patients with periappendiceal abscesses should be treated with percutaneous image-guided drainage**,** few patients underwent this procedure.

The laparoscopic versus open cholecystectomy debate has been extensively investigated in recent years. In the CIAO Study, the open cholecystectomy was the most common means of treating cholecystitis; 48.4% of patients with complicated cholecystitis underwent this procedure. By contrast, 118 patients (40.8%) underwent the laparoscopic procedure.

The optimal surgical management of colonic diverticular disease complicated by peritonitis remains a controversial issue in the medical community.

Hartmann’s resection has historically been considered the procedure of choice for patients with generalized peritonitis and continues to be a safe and reliable technique for performing an emergency colectomy in the event of perforated diverticulitis, particularly in elderly patients with multiple co-morbidities [7-10].

More recently, however, reports have suggested that primary resection and anastomosis may be the optimum approach to addressing diverticulitis, even in the presence of diffuse peritonitis [11].

According to CIAO Study data, the Hartmann resection was the most frequently performed procedure to address complicated diverticulitis in Europe. 43.2% of patients underwent a Hartmann resection, and of these resections, the vast majority were open procedures (94.5% open compared to 5.5% laparoscopic). 54 of these patients (74%) underwent a Hartmann resection for generalized peritonitis, while the remaining 19 (26%) underwent the same procedure for localized peritonitis or abscesses.

22.5% of patients underwent colo-rectal resection to address complicated diverticulitis.

### Microbiology

The significance of microbiological analysis of infected peritoneal fluid in community-acquired intra-abdominal infections has been debated in recent years.

Cultures from the site of infection should always be obtained for patients with nosocomial infections as well as for patients with community-acquired infections who are known to be at risk for drug-resistant strains. In these patients, causative pathogens and resistance patterns are unpredictable and always require cultures from the site of infection [4].

Bacterial cultures and analyses may be often clinically superfluous, particularly when the etiological agents are readily predictable [12]. However, some authors maintain that in-depth bacterial diagnosis has practical significance, even in low-risk patients with community-acquired IAIs. They argue that this analysis plays an important role in documenting epidemiological shifts in antimicrobial resistance patterns associated with community-acquired IAIs and in guiding individualized follow-up therapy. For high-risk patients with community-acquired IAIs or in the event of nosocomial IAIs, clinicians should always obtain cultures from the site of infection.

According to CIAO Study data, intraperitoneal specimens were collected from 62.2% of patients; these samples were obtained from 57.4% of patients with community-acquired IAIs and from 80.3% of patients with nosocomial IAIs.

In many clinical laboratories, species identification and susceptibility testing of anaerobic isolates are not routinely performed [13].

Of the total patients tested for aerobic microorganisms, 42.9% underwent tests for anaerobes.

The major pathogens involved in community-acquired intra-abdominal infections are *Enterobacteriaceae*, *Streptococcus sp*ecies, and certain anaerobes (particularly *B. fragilis*). Compared to community-acquired infections, nosocomial infections typically involved a broader spectrum of microorganisms, encompassing ESBL-producing *Enterobacteriaceae*, *Enterococcus, Pseudomonas,* and *Candida* species in addition to the *Enterobacteriaceae, Streptococcus species,* and anaerobes observed in community-acquired IAIs.

Antimicrobial resistance has become a major challenge complicating the treatment and management of intra-abdominal infections.

The main resistance threat is posed by ESBL-producing *Enterobacteriaceae*, which are becoming increasingly common in community-acquired infections.

Many factors can increase the prevalence of ESBL activity in community-acquired intra-abdominal infections, including excessive use of antibiotics, residence in a long-term care facility, and recent hospitalization. Further, male patients and patients over the age of 65 appear to be particularly susceptible to ESBL-producing bacterial infections [14].

According to CIAO Study data, ESBL producers were the most commonly identified drug-resistant microorganism involved in IAIs.

Recent years have seen an escalating trend of Klebsiella pneumoniae Carbapenemase (KPC) production, which continues to cause serious multidrug-resistant infections around the world. The recent emergence of Carbapenem-resistant *Enterobacteriaceae* is a major threat to hospitalized patients.

In addition to hydrolyzing Carbapenems, KPC-producing strains are also resistant to a variety of other antibiotics, and consequently, these infections pose a considerable challenge for clinicians in acute care situations.

KPC-producing bacteria are most common in nosocomial infections, particularly in patients with previous exposure to antibiotics [15].

5 identified isolates of *Klebsiella pneumoniae* proved resistant to Carbapenems, and each was acquired in an intensive care setting.

The rate of *Pseudomonas aeruginosa* among aerobic isolates was 5.2%. There was no statistically significant difference in *Pseudomonas* prevalence between community-acquired and nosocomial IAIs.

*Enterococci* (*E. faecalis and E. faecium*) were identified in 15.7% of all aerobic isolates.

Although *Enterococci* were also identified in community-acquired infections, they were far more prevalent in nosocomial infections.

In the CIAO Study, 138 *Candida* isolates were observed among 1,890 total isolates (7.3%). *Candida* prevalence was significantly higher in the nosocomial IAI group than it was in the community-acquired IAI group.

### Outcome

Of the 2,152 patients enrolled in the study, there were 163 deaths (7.6%).

According to univariate statistical analysis of the data, critical clinical condition of the patient upon hospital admission (defined by severe sepsis/septic shock) as well as critical clinical condition in the immediate post-operative period and ICU admission were all significant risk factors predictive of patient mortality. WBCs greater than 12,000 or less than 4,000 and core body temperatures greater than 38°C or less than 36°C by the third post-operative day were predictors of patient mortality. Among the various sources of infection, colonic non-diverticular perforations, complicated diverticulitis, and small bowel perforations correlated strongly with patient mortality.

Mortality rates did not vary to a statistically significant degree between patients who received adequate source control and those who did not. However, a delayed initial intervention (a delay exceeding 24 hours) was associated with an increased mortality rate.

According to stepwise multivariate analysis (PR=0.005 and PE=0.001), several criteria were found to be independent variables predictive of patient mortality, including patient age, the presence of an intestinal non-appendicular source of infection (colonic non-diverticular perforation, complicated diverticulitis, small bowel perforation), a delayed initial intervention (a delay exceeding 24 hours), sepsis and septic shock in the immediate post-operative period, and ICU admission.

## Conclusion

Complicated intra-abdominal infections remain an important source of patient morbidity and are frequently associated with poor clinical prognoses, particularly for patients in high-risk categories.

Given the sweeping geographical distribution of the participating medical centers, the CIAO Study gives an accurate description of the epidemiological, clinical, microbiological, and treatment profiles of complicated intra-abdominal infections (IAIs) throughout Europe.

## Competing interests

The authors declare that they have no competing interests.

## Authors’ contributions

MS designed the study and wrote the manuscript. FC, LA, AL, KT, HVG, DVL, PV and CDW participated in study design. DVL revised the manuscript. FCo and DC performed statistical analysis. All authors read and approved the final manuscript.
